# Influence of dance therapy mode of intangible cultural heritage on children's emotional regulation and psychological wellbeing: a randomized controlled intervention study

**DOI:** 10.3389/fpubh.2026.1896823

**Published:** 2026-08-31

**Authors:** Xuetao Liu, Shuwei Xie

**Affiliations:** 1 Tianjin University of Sport, Tianjin, China; 2 Hanyang University, Seoul, Republic of Korea

**Keywords:** children's mental health, dance therapy, emotional regulation, intangible cultural heritage dance, psychological intervention, social adaptability

## Abstract

**Background:**

Children's psychological problems frequently appear, and the exploration of coping schemes without drugs, physical and mental integration and cultural depth has attracted academic attention. The intangible dance contains rhythmic movements, group cooperation, emotional expression and cultural implications, which make it a powerful tool for psychological intervention. The research on the educational application of dance in the field of intangible cultural heritage mainly focuses on conventional art therapy and music intervention. It is still a relatively new research direction to apply dance to children's mental health.

**Target:**

In this study, an orderly framework of dance therapy for intangible cultural heritage was built. This framework examines its effects on children's emotional regulation, anxiety, signs of depression, self-worth and social adaptability, and explores the actual impact and mechanism of this treatment model.

**Way:**

In this study, a randomized comparative test program was carried out. A total of 100 children aged 6 to 12 with mild emotional sensitivity, social maladjustment, increased stress level or attention disorder were randomly assigned to the experimental group (receiving ICH dance therapy intervention) and the control group (receiving routine psychological group counseling), and each group included 50 subjects. The intervention period is 16 weeks, including 12 weeks of structured dry expectation and 4 weeks of follow-up observation, so the implementation of intervention measures will be carried out three times a week, and the time will be maintained in the range of 35–45 min. The intervention scheme used in this study is composed of six links, including rhythmic exercise, emotional expression dance, rhythm relaxation exercise, collective dance interaction, cultural element integration and self-emotional examination. Emotional detection covers adjustment, anxiety level (GAD-7), depression score (PHQ-9), self-esteem status, social integration, participation consistency and emotional stability standards. In the field of data analysis, the implementation of repeated measurement analysis of variance is carried out, and the correlation is discussed with the help of Pearson correlation coefficient.

**Result:**

After carrying out the experimental program, the members of the treatment group showed better promotion compared with the reference group in many aspects. Its emotional management has been improved, anxiety has been reduced, depression has been alleviated, self-worth has been enhanced, and social integration ability has also improved. Statistical tests have proved that these differences are significant. The study observed significant time effect and interaction effect between groups and time, and the improvement of emotional adjustment ability was positively correlated with the improvement of social adaptability and self-esteem, and the correlation coefficient was greater than 0.70, and the statistical test results were significant. There was no obvious bad mood or physical symptoms in the intervention stage.

**Conclusion:**

The dance therapy mode of intangible cultural heritage has a significant positive impact on children's emotional adjustment ability and psychological and social development. This intervention will integrate physical activities, emotional release, group participation and cultural identity to enhance children's mental health, and open up a non-medical intervention path with structure, safety and cultural connotation. This study provides the necessary theoretical basis and practical support for the integration of mental health cultivation, aesthetic cultivation and the persistence of intangible cultural heritage in the child growth intervention system.

## 1 Introduction

Childhood is a key stage of development in emotional cognition, personality formation, social interaction and psychological resilience construction. According to the theoretical framework constructed by Piaget and Eriksson, children aged 6–12 are entering a stage of rapid development in their self-cognition construction, interpersonal interaction ability and behavior regulation mechanism. Children's emotional control and external adaptation are usually regarded as two key references to measure their psychological state and social integration (1). Specifically, the ability of emotional control means that individuals can feel, judge, express and adjust their emotional experience to meet the various requirements put forward by the environment (2, 3); However, the effectiveness of individuals in dealing with social affairs is usually reflected in the comprehensive level of building interpersonal networks, participating in group exchanges and maintaining the coordination of words and deeds in the community structure (4).

In recent years, children's inner state has become more and more worrying all over the world, and the rising trend is very noticeable (5). The report reveals that school-age minors have multiple emotional problems, and the monitoring system has the function of providing such data. Children's mental health has aroused widespread concern, focusing on negative psychological performance. These phenomena have appeared in many countries, highlighting the universality of this problem, so emotional acuity and avoidance behavior in social interaction have become more and more common (6). The National Mental Health Development Report and various epidemiological surveys have consistently revealed that the occurrence of emotional and behavioral disorders is spreading to younger groups, and such signs have become more and more clear in China. Excessive exposure to digital media and the decline in physical activity will weaken children's social interaction. The pressure of family environment and the decrease of interaction with peers will also aggravate their vulnerability in emotional development. Speech counseling and cognitive education have always been the core path to carry out psychological intervention. However, in the process of stimulating children's active participation, deepening their emotional involvement and maintaining continuous cooperation, these methods often show shortcomings. Children's intervention projects need to give priority to the characteristics of children's growth, and it is necessary to integrate the direct perception of limbs and the emotional blending response, so it is necessary to carry out such practice immediately.

Dance therapy has gradually attracted the attention of academic circles, mainly because it combines body rhythm (7), emotional expression and social communication, thus forming an innovative non-drug intervention approach (8–10). Dance therapy closely links psychological experience with physical action, and its core point of view is that choreographed dance movements can make internal emotions experience explicit, transform and control (11). Dancing therapy can change the emotional, social and psychological resilience problems of children and adolescents. The intervention method with body as the carrier can start embodied cognition (12, 13), emotional resonance, mirror neuron network and emotional regulation path, and then promote the growth of comprehensive psychology (14).

Among all kinds of intervention forms based on sports, intangible cultural heritage dance has unique therapeutic potential. ICH dance is an unusual exercise and entertainment activity, which contains multiple elements such as rhythm coordination, symbolic gesture, group blending, emotional resonance, ritual communication and cultural identity shaping. Ancient dance carries cultural memories and emotions, from which children can release their emotions, improve their cooperation ability and aesthetic perception, and enhance their sense of belonging to cultural groups. Then the rhythm of dancing will repeatedly adjust their autonomic nerves and change their emotional stability (15). The interactive nature of group dance virtually promotes the communication between individuals and the integration of social environment.

The intervention methods of artistic healing and practical exercise orientation are gradually attracting more and more attention, but there are still multiple boundaries in the previous exploration, and the research on non-legacy dance therapy is rarely carried out. Most of the existing intervention studies focus on music therapy, painting therapy and regular sports activities; In the previous investigations, there were few unified intervention methods, and there was a lack of experimental framework for random distribution and long-term follow-up monitoring. As a result, the reliability and application scope of the research conclusions were limited. It is rarely carried out to explore several aspects of children's psychological development together with the analysis of the framework intervention model, that is, regulating emotions, anxiety, depression, self-esteem and social adaptation; The psychological mechanism of cultural internalized dance intervention on children's mental health needs further discussion.

At present, educational innovation has been paying constant attention to aesthetic cultivation, conscious learning and the continuation of cultural heritage. If intangible dance is included in the category of children's psychological healing, it is expected to realize a new way to promote mental health and cultural education. This study attempts to build a framework system based on intangible cultural heritage dance therapy, and uses random control method to test its intervention effect on children's emotional self-regulation, anxiety and depression, which affects their self-esteem and social adaptability (16, 17). The theoretical framework of dance therapy and embodied intervention has been enriched, which provides an empirical basis for integrating intangible cultural heritage resources into children's mental health education and its application in school psychological intervention system.

## 2 Methods

### 2.1 Research design and ethical considerations

The Ethics Committee of Tianjin Sport University approved this research, namely TJUS 2026_045. This approval work is carried out entirely by virtue of the moral standards established in the Helsinki Declaration.

The legal guardian signed a written consent document before joining the group, and the researchers specially carried out the corresponding consent process in view of different age groups and understanding ability.

Therefore, structured intervention measures are formulated to greatly reduce the potential risks in psychology and physiology. Physical activity and emotional intensity need to be kept within a safe threshold, and participants have the right to stop the research at any time without being punished. During the whole research process, the subjects' information was carefully protected, and their privacy and anonymity were also maintained.

The evaluation of project closure will be carried out by special evaluators, who will not participate in the intervention operation and will not know the situation of subjects grouping during the whole information collection stage.

During this evaluation stage, participants and instructors are not allowed to exchange the assignment details of the group.

The work of anonymous processing of data has been carried out. In view of this encoded data set, the analysis work can be carried out, which can ensure that the test deviation is reduced.

### 2.2 Participants

The selection process will cover community child development centers and primary schools, and the selection targets are children aged 6–12. There are 100 eligible children who will participate by voluntary registration and psychological screening.

#### 2.2.1 Inclusion criteria

Based on the research rules, some people are included in the scope of investigation.

1. Carry out the work of positioning the core age group in the middle of childhood;
2. It is characterized by mild emotional sensitivity, social maladjustment, increased stress level, attention disorder or emotional instability;
3. Physique has the ability to withstand moderate intensity exercise;
4. No systematic dance therapy training experience;
5. The guardian personally carries out the approval of the notification and consent documents.

#### 2.2.2 Exclusion criteria

If the people involved in the experiment have any specified situation, they will be removed from the group.

6. Suffering from serious mental illness and developmental problems;
7. Severe attention deficit hyperactivity disorder (ADHD);
8. The impairment of motor ability is usually triggered by central nervous system diseases;
9. Some people are undergoing psychological counseling and intervention with mental health drugs;
10. In view of the relatively high physical requirements of related activities, it is difficult for individuals to participate in moderate-intensity sports due to health reasons.

#### 2.2.3 Randomization and group allocation

The intervention of non-legacy dance therapy was given to the experimental group, and the qualified personnel were randomly assigned to the experimental group, that is, *n* = 50, or the control group, that is, *n* = 50, and the traditional group psychological counseling was carried out in the comparison group. With the help of computer, the randomization process can be generated, so as to realize the division of groups. The baseline characteristic data of the two groups can be compared, such as age, gender, emotional control, self-esteem, anxiety, depression and social adaptation. No significant differences are observed statistically, and all *p*-values exceed 0.05. In the initial state, the basic characteristics between the two groups show sufficient similarity, which makes the subsequent comparison have a reliable basis.

### 2.3 Intervention plan

#### 2.3.1 General intervention framework

This intervention will last for 12 weeks, and three courses will be arranged every week, each course will last for about 40 min. Dance coaches and psychological counselors will work together to carry out the intervention work in a specific room, that is, a safe space, and give professional guidance. In order to ensure the safety of participants and meet the ethical requirements, the intervention intensity will be limited to a low to medium level, the emotional stimulation will be strictly controlled, and at the same time, the competitive or high-pressure activities will be avoided, then the emotional state of participants will be continuously concerned, and the exit procedure will be ready to start at any time to deal with negative emotions.

#### 2.3.2 ICH dance therapy intervention

Traditional dance elements constitute six components of non-legacy dance therapy, that is, rhythm awakening, emotional action display and celebration theme activities. After the interactive integration of group dance, people's body and mind can be relieved through this activity, and cultural cognition will also be enhanced.

The content of the intervention is aimed at children aged 6–12, so as to fit their growth stage, protect their psychological safety, and encourage them to take the initiative to participate in it. The focus of the class is on emotional expression, social interaction and physical civilization experience, while the skill display will not be placed at the core.

This intervention program integrates the characteristics of typical children's Chinese folk dance, and incorporates Tibetan dance, Mongolian dance style, dai dance rhyme and Uygur dance.

The selected evaluation mechanism highlights the rhythm permission, emotional transmission, cultural symbol value, adaptability to the development stage, and attention to the safety of school-age children.

Embodied expression focuses on emotional expression, and social interaction and cultural integration have been improved, which goes beyond the simple dance technique display.

#### 2.3.3 Control group: routine psychological counseling

Children in the control group will receive routine psychological group counseling.

The intervention plan of the control group takes the same arrangement as that of the experimental group as the use, and carries out treatment three times a week, each treatment lasts for 35–45 min, so as to ensure comparability under different conditions.

Consulting activities include emotional cognitive training, guided relaxation training, verbal communication practice and structured social interaction tasks. Then, it does not cover dance movements, rhythmic body expression and intangible cultural heritage elements.

In order to make the intervention intensity between different groups more referential, the frequency of intervention behavior, the implementation length and the total project span of each group are set to be roughly the same. Under the guidance of the group, the curriculum framework will be run in the way after arrangement.

After the children were divided into the control group, they participated in structured psychological guidance activities in a small group environment, in which each group contained about 8–10 people.

Then, well-trained personnel will take the standardized scheme as a criterion to carry out the practice of diagnosis and treatment process.

The exposure of intervention obtained by the experimental group is similar to that of the control group in terms of frequency, duration and whole cycle.

### 2.4 Outcome indicators

Then children will accept and match their age assessment methods.

Guardians can independently evaluate children's emotional adjustment and social adaptability by daily observation.

In the process of children's self-assessment, the researchers carried out guidance work on the spot, and the selected scales included GAD-7 and PHQ-9, with the help of this interview form to ensure the authenticity and objectivity of the assessment.

From the historical investigation, it is observed that children and teenagers show the stability and accuracy of using the measurement method.

#### 2.4.1 Main outcome indicators

##### 2.4.1.1 Emotional adjustment ability

Emotional adjustment ability can be evaluated from the level of children, and it will be measured by ERC scale. The dimensions involved in this scale include emotional cognition, emotional expression and emotional adaptation.

##### 2.4.1.2 Anxiety symptoms

Generalized anxiety symptoms will be observed with seven self-assessment tools.

##### 2.4.1.3 Depressive symptoms

Depressive tendency was assessed by Patient Health Questionnaire-9 (PHQ-9).

##### 2.4.1.4 Social adaptation ability

In this assessment, the children and adolescents adaptation scale (CASAS) is selected to consider its performance in team communication, rule compliance and environmental response.

#### 2.4.2 Secondary outcome indicators

Secondary indicators include:

Self-esteem level;
Participation compliance rate;
Frequency of emotional participation;
Frequency of group cooperation;
Cultural identity cognition;
Emotional changes reported by parents;
Social interaction of teachers' reports.

#### 2.4.3 Safety monitoring indicators

During the intervention stage, continuous attention will be paid to these key safety parameters:

The occurrence of emotional distress;
Fatigue and discomfort;
The possibility of sports injury will rise accordingly;
Withdrawal rate;
Emotional overload reaction.

At this stage of the trial, there were no serious adverse reactions.

### 2.5 Statistical analysis

Data were analyzed by SPSS 27.0 software (IBM Corporation, Armonk, USA).

The measured value takes the form of average plus or minus standardized deviation, and its expression paradigm is X plus or minus SD. The presentation of classified variables will involve frequency statistics and proportional calculation.

Statistical analysis will be carried out according to the following process:

11. Independent sample *t*-test for baseline comparison;
12. The variance analysis method of repeated measurement is used to carry out intra-group and inter-group comparison. This analysis is carried out in order to evaluate the differences before and after itself and between different groups at the same time;
13. One-way ANOVA was used to compare between groups.
14. With the help of Pearson correlation analysis, it evaluates the relationship between emotional regulation mechanism and various indicators of psychosocial category;
15. The analysis of partial η effect is carried out.

*P*-value < 0.05 is considered statistically significant.

## 3 Results

### 3.1 Baseline characteristics

All the participants recruited in this experiment have accepted the initial evaluation work. After the comparison between the experimental group and the control group, there is no significant difference in age and gender data, the difference in baseline emotional adjustment score is not obvious, the difference in anxiety and depression score is not obvious, and the difference in social adaptability score is not obvious. After the comparison of the data of each group, the *P*-value is greater than 0.05, which constitutes the basis for matching reference. The demographic data and baseline psychological indicators of the subjects are shown in Table 1.

**Table 1:** Lists the demographic characteristics and baseline psychological indicators of the subjects.

Variable	Experimental group (*n* = 50)	Control group (*n* = 50)	Probability value
Age (years)	8.73 ± 1.42	8.65 ± 1.37	0.742
Gender (male/female)	26/24	25/25	0.841
GAD-7 score	12.46 ± 2.31	12.38 ± 2.27	0.887
PHQ-9 score	11.22 ± 2.08	11.15 ± 2.14	0.901
ERC emotion regulation score	42.51 ± 5.27	43.02 ± 5.34	0.673
Social adaptability score	48.33 ± 6.11	47.92 ± 6.25	0.781
Self-esteem score	21.84 ± 3.17	21.56 ± 3.24	0.694

### 3.2 Comparison of emotional adjustment ability before and after intervention

**Figure 1:**
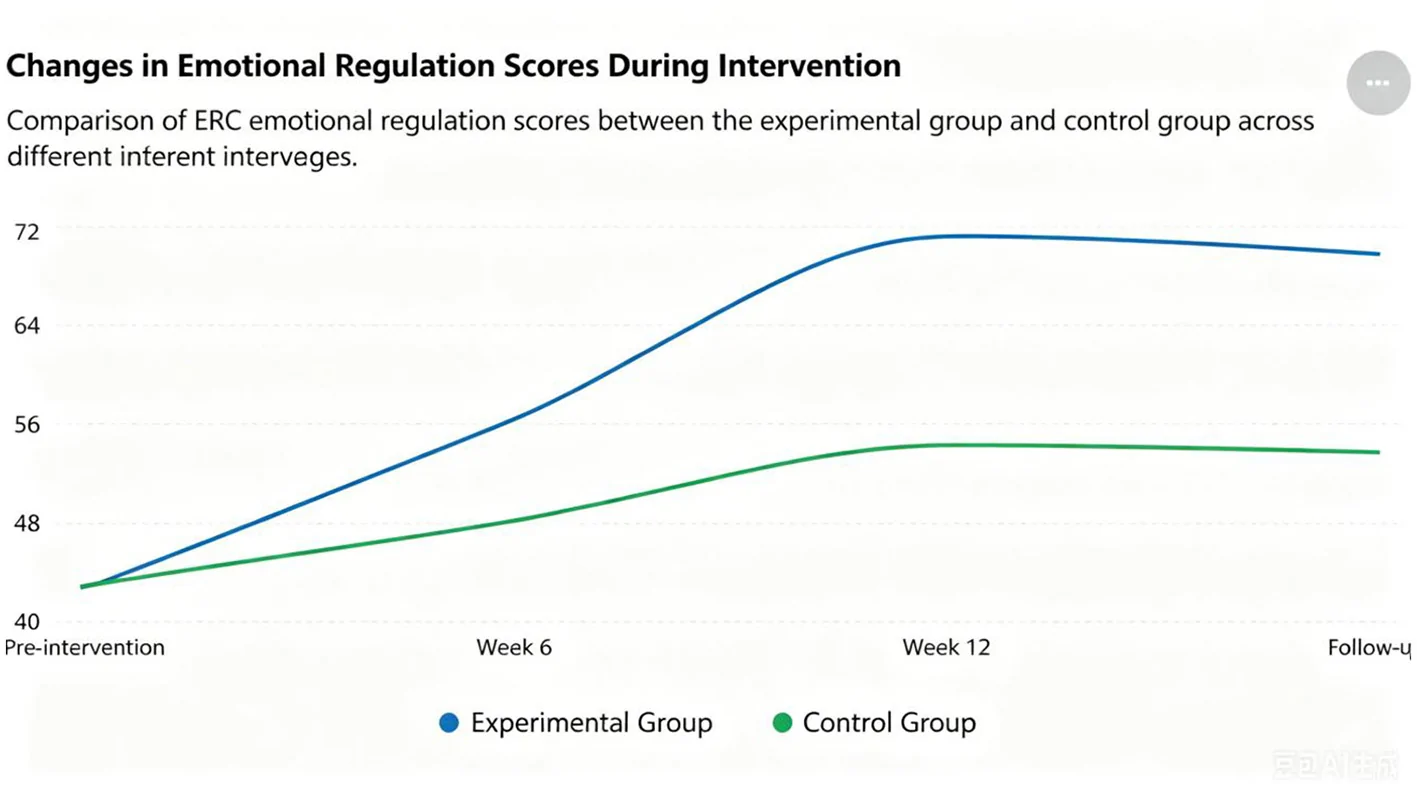
Changes in Emotion Regulation Checklist (ERC) scores in the experimental and control groups at pre-intervention, week 6, week 12, and follow-up.

After 3 weeks of scientific research intervention, the emotional management skills of the observation group were observed and found to be obviously optimized compared with the control group, and the results of statistical test were significant, and the confidence interval was fully considered. The changes in ERC scores of the two groups at pre-intervention, week 6, week 12 and follow-up are shown in Figure 1.

In all dimensions:

In fact, the extent of emotional exposure has reached its peak;
The process of emotion regulation by cognition has been significantly improved;
The emotional stability score continued to rise steadily.

Then the time effect is significant, and the interaction with the group is also obvious, which is confirmed by repeated measurement and analysis.

### 3.3 Comparison of anxiety and depression indicators

**Figure 2:**
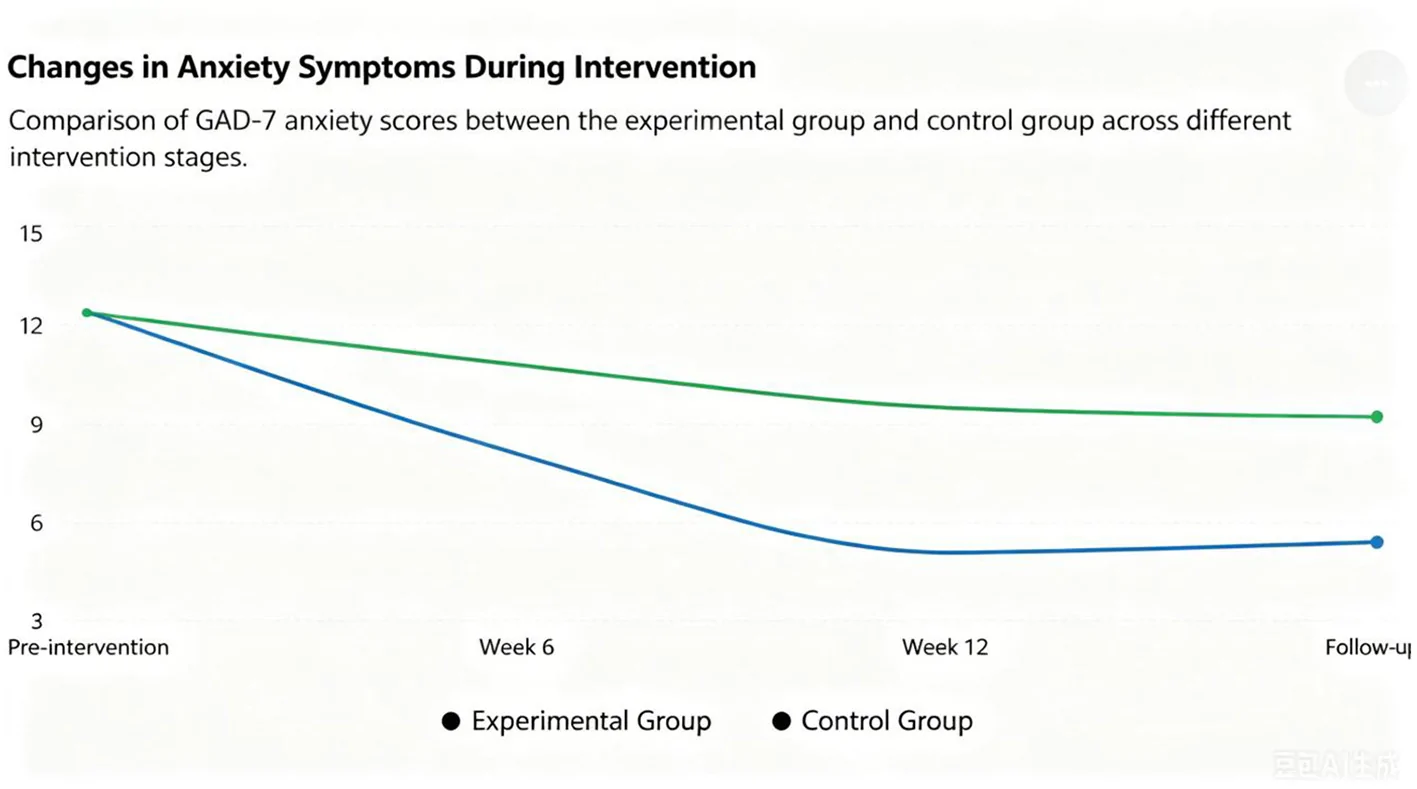
Changes in GAD-7 anxiety scores in the experimental and control groups at pre-intervention, week 6, week 12, and follow-up.

Taking the baseline value as a reference, both groups of subjects showed the relief of anxiety and depression symptoms after intervention; compared with the control group, the improvement performance of the experimental group will be more significant. The dynamic changes in GAD-7 anxiety scores of the two groups at pre-intervention, week 6, week 12 and follow-up are shown in Figure 2, and the comparison of psychological indicators before and after intervention is presented in Table 2.

**Table 2:** Comparison of psychological indexes before and after intervention.

Variable	Time	Experimental group (*n* = 50)	Control group (*n* = 50)	Probability value
ERC emotion regulation score	Before intervention	42.51 ± 5.27	43.02 ± 5.34	0.673
	After the intervention,	71.34 ± 6.12	54.28 ± 5.94	< 0.001
GAD-7 score	Before intervention	12.46 ± 2.31	12.38 ± 2.27	0.887
	After the intervention,	5.12 ± 1.84	9.46 ± 2.11	< 0.001
PHQ-9 score	Before intervention	11.22 ± 2.08	11.15 ± 2.14	0.901
	After the intervention,	4.83 ± 1.56	8.74 ± 1.97	< 0.001
Social adaptability score	Before intervention	48.33 ± 6.11	47.92 ± 6.25	0.781
	After the intervention,	74.85 ± 6.73	58.21 ± 5.96	< 0.001
Self-esteem score	Before intervention	21.84 ± 3.17	21.56 ± 3.24	0.694
	After the intervention,	33.42 ± 3.65	25.74 ± 3.11	< 0.001

Each group of the subjects showed the observed specific characteristics.

GAD-7 score decreased significantly;
PHQ-9 score decreased significantly;
Individuals' ability to carry out emotional regulation and control over negative situations and their tenacity have been significantly improved.

The observation group only showed a moderate improvement.

### 3.4 Comparison of social adaptability

**Figure 3:**
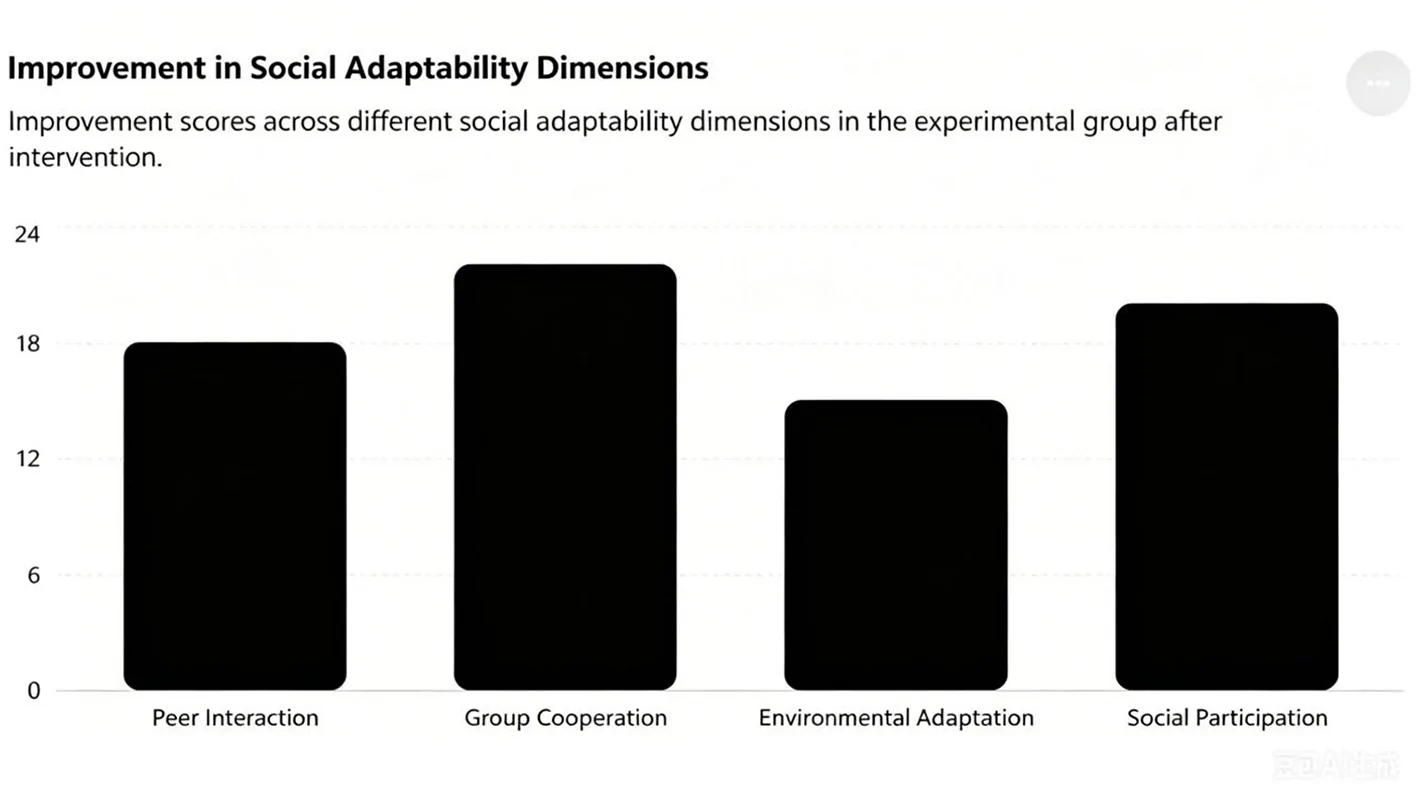
Improvement of social adaptability dimension.

The improvement of the children's groups in several dimensions is particularly obvious. The improvements in each dimension of social adaptability are shown in Figure 3.

Peer interaction;
Group cooperation;
Social participation;
Environmental adaptation;
Collective integration.

Interaction and rhythmic cooperation will mobilize children's social interaction in dance and synchronize their emotional trends.

### 3.5 Correlation analysis

**Figure 4:**
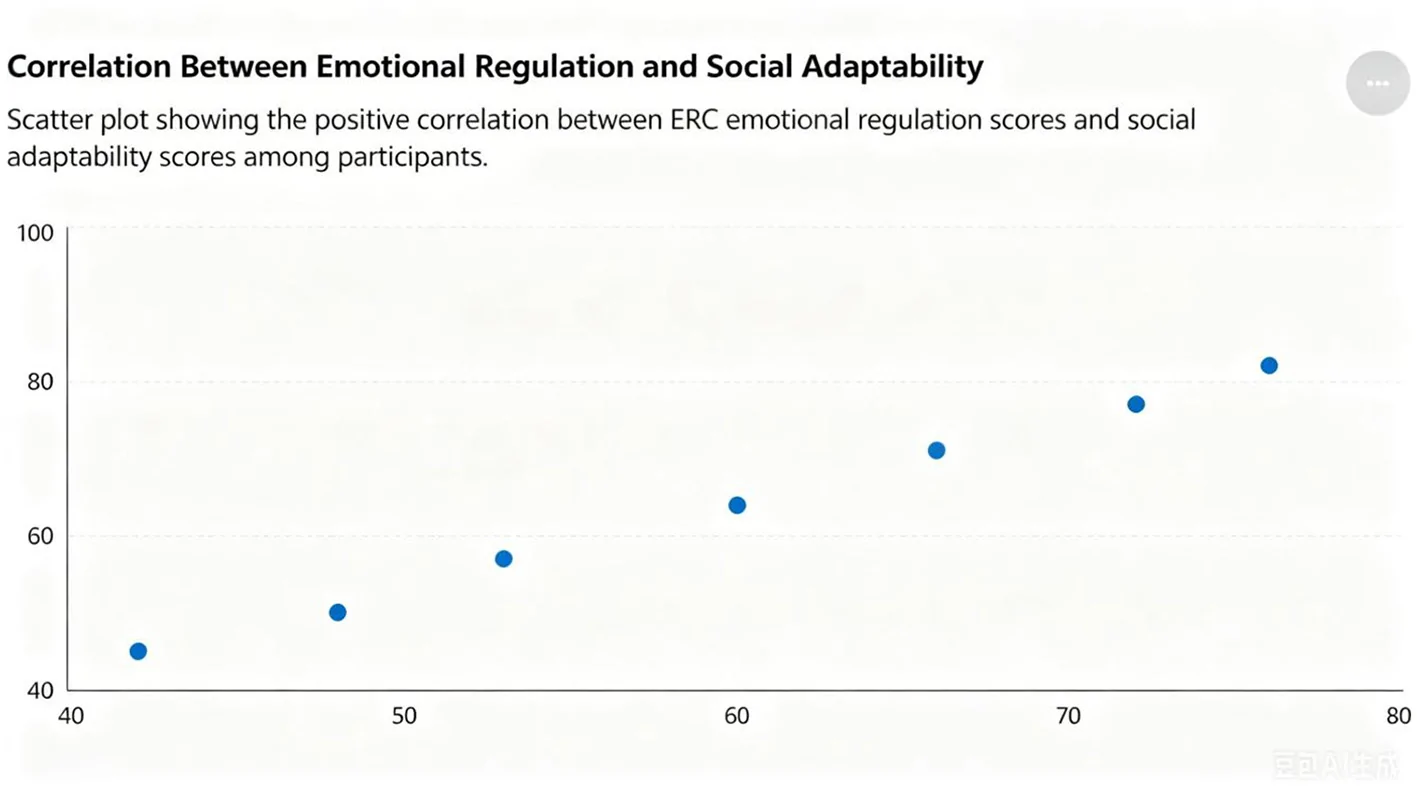
Correlation between emotional regulation and social adaptability.

Pearson correlation analysis is used to carry out the analysis, which reveals that there is a positive correlation between emotional regulation ability and specific parameters. The correlation between emotional regulation and social adaptability is shown in Figure 4, and the repeated-measures ANOVA results of psychological indicators are presented in Table 3.

Social adaptability (*r* > 0.70, *P* < 0.001);
Their respect attitude shows a high continuous correlation, that is, the correlation strength is greater than 0.65, and the statistical significance *P*-value is lower than 0.001;
Participation frequency (*r* > 0.60, *P* < 0.01).

**Table 3:** Results of repeated measurement variance analysis of psychological indicators.

Variable	Time effect (f)	Group effect (f)	Interaction effect (f)	Probability value
ERC emotion regulation score	84.32	56.71	49.28	< 0.001
GAD-7 score	71.54	42.83	38.62	< 0.001
PHQ-9 score	68.17	40.25	35.71	< 0.001
Social adaptability score	77.46	51.38	43.16	< 0.001
Self-esteem score	63.28	39.44	32.08	< 0.001

Such findings suggest that if the ability of emotional regulation is strengthened, it may promote a wider range of psychological and social development.

## 4 Discussion

This study confirmed that the intangible cultural heritage dance therapy model significantly improved the emotional adjustment ability, anxiety symptoms, depression tendency, self-esteem level and social adaptability of children aged 6–12. Compared with the conventional way of psychological counseling, dance intervention, a new program, will achieve more remarkable results in improving emotional state and social psychology.

The manipulation of children's emotions is carried out with the help of embodied sports (18). Body rhythm stimulates brain regulation (19). According to the theory of embodied cognition, emotional experience is closely related to body movement and sensory perception. Repetitive rhythm, coordinated movements and breathing synchronization in dance can help to stabilize the autonomic nervous system and realize emotional self-regulation. Children realize the gradual accumulation of emotional awareness and emotional management literacy by means of continuous body-rhythm interaction (20, 21).

The mechanism of emotional externalization may include symbolic expression, and it is often difficult for children to explain complex emotions with words (22). As a communication channel of non-verbal emotions, dance movements can make children externalize emotions such as tension, anxiety, fear and excitement with the help of body movements. In this study, dance is introduced as a form of emotional expression. This arrangement is conducive to the open expression of emotions and the suppression of emotions can be alleviated (23).

The cooperative group structure of ICH dance may also explain its remarkable effect in improving social adaptability, so the cohesion of common melody requires children to continuously observe the emotions of their partners and carry out the coordination of interpersonal communication to cultivate community identity. Synchronized actions, such as coordinated behaviors among groups, can strengthen the trust between people, enhance the ability of empathy and enhance the sense of belonging of the group, which is clearly confirmed in the conclusion of this study (24).

In addition, the cultural characteristics contained in ICH dance may bring unique psychological benefits beyond ordinary physical exercise. The ancient dance marks the cultural context and expresses the moral and aesthetic consensus. Participating in specific activities with rich cultural significance can strengthen children's attachment to culture, improve children's positive feelings about self-identity and enhance their emotional stability (25, 26). The harmonious combination of culture and emotion makes the presentation of dance therapy unique and clearly distinguishes it from common entertainment and leisure activities.

Then, before the screening, the participants have fulfilled the qualification and ethical procedures, but the standardized neuropsychological measurement is absent in the basic evaluation stage, so it is difficult for researchers to fully manipulate the diversity of individuals in development and cognitive characteristics (27). Future research can include a wider range of diagnostic methods and developmental testing programs, and continue to extend the follow-up period, in order to achieve the universal applicability of intervention effectiveness and enhance the significance of long-term evaluation (28).

## 5 Conclusion

Dance therapy, with its intangible elements, has a clear effect on improving children's mood, thus promoting the psychological development process (29). Compared with conventional psychological counseling, this intervention method can make emotional speech expression smoother, deal with the work of reducing anxiety experience, relieve low emotions, achieve self-affirmation and improve the efficiency of interpersonal adjustment (30).

After rhythmic movements combine emotional expression and cooperative cognition, dance has evolved into a structural body and mind promotion system. This kind of intervention can not only help psychological adjustment, but also have the dual function of cultural education. This study provides theoretical support and practical basis for integrating intangible cultural heritage resources into children's mental health intervention system, aesthetic education reform and school mental health projects.

## Data Availability

The original contributions presented in the study are included in the article/supplementary material, further inquiries can be directed to the corresponding author.

## References

[1] Yilmazer E. Emotional regulation in adolescence: a comprehensive review. Nişantaşi Üniversitesi Sosyal Bilimler Dergisi. (2024) 12:403–24. doi: 10.52122/nisantasisbd.1457804

[2] Gross JJ. Emotion regulation: current status and future prospects. Psychol Inq. (2015) 26:1–26. doi: 10.1080/1047840X.2014.940781

[3] Eisenberg N Spinrad TL. Emotion-related regulation: sharpening the definition. Child Dev. (2004) 75:334–9. doi: 10.1111/j.1467-8624.2004.00674.x 15056187

[4] Denham SA. Emotional competence and social adjustment in childhood. Child Dev Perspect. (2007) 1:24–30.

[5] World Health Organization. Mental Health of Children and Adolescents. Geneva: World Health Organization (2024).

[6] UNICEF. The State of the World's Children 2021: On My Mind—Promoting, Protecting and Caring for Children's Mental Health. New York, NY: United Nations Children's Fund (2021).

[7] Bräuninger I. Dance Movement Therapy Group Intervention in Stress Treatment: A Randomized Controlled Trial (RCT). The Arts in Psychotherapy. (2012) 39:443–50. doi: 10.1016/j.aip.2012.07.002

[8] KochSC RiegeRFF TisbornK BiondoJ MartinL BeelmannA. Effects of dance movement therapy and dance on health-related psychological outcomes: a meta-analysis. Arts Psychother. (2019) 63:72–84. doi: 10.3389/fpsyg.2019.0180631481910 PMC6710484

[9] KarkouV AithalS ZubalaA MeekumsB. Effectiveness of dance movement therapy in the treatment of adults with depression: a systematic review. Front Psychol. (2019) 10:936. doi: 10.3389/fpsyg.2019.0093631130889 PMC6509172

[10] KochSC KunzT LykouS CruzR. Effects of dance movement therapy and dance on health-related psychological outcomes: a meta-analysis update. Arts Psychother. (2014) 41:46–64. doi: 10.1016/j.aip.2013.10.00431481910 PMC6710484

[11] Meekums B. Dance Movement Therapy: A Creative Psychotherapeutic Approach. London: Sage Publications (2002). doi: 10.4135/9781446217986

[12] Shapiro L. Embodied Cognition. 2nd ed. London: Routledge (2019). doi: 10.4324/9781315180380

[13] Wilson M. Six views of embodied cognition. Psychon Bull Rev. (2002) 9:625–36. doi: 10.3758/BF03196322 12613670

[14] Malchiodi CA. Creative Arts Therapy and Children. New York, NY: Guilford Press (2005).

[15] Saarikallio S. Music as emotional self-regulation throughout adulthood. Psychol Music. (2011) 39:307–27. doi: 10.1177/0305735610374894

[16] Orth U Robins RW. The development of self-esteem. Curr Dir Psychol Sci. (2014) 23:381–7. doi: 10.1177/0963721414547414

[17] Rosenberg M. Society and the Adolescent Self-Image. Princeton, NJ: Princeton University Press (1965). doi: 10.1515/9781400876136

[18] Schore AN. Affect Regulation and the Origin of the Self: The Neurobiology of Emotional Development. New York, NY: WW Norton & Company (1994).

[19] Payne H. The body speaks its mind: the body as a bridge between conscious and unconscious processes. Body, Mov Dance Psychother. (2006) 1:7–21. doi: 10.1080/17432970500468117

[20] Koch SC. Arts and health: active factors and a theory framework of embodied aesthetics. Arts Psychother. (2017) 54:85–91. doi: 10.1016/j.aip.2017.02.002

[21] PorgesSW. The polyvagal perspective. Biol Psychol. (2007) 74:116–43. doi: 10.1016/j.biopsycho.2006.06.00917049418 PMC1868418

[22] TarrB LaunayJ DunbarRIM. Music and social bonding: self–other merging and neurohormonal mechanisms. Front Psychol. (2014) 5:1096. doi: 10.3389/fpsyg.2014.0109625324805 PMC4179700

[23] McMahon SD Wernsman J Parnes AL. Understanding prosocial behavior: the impact of empathy and gender among African American adolescents. J Adolesc Health. (2006) 39:135–7. doi: 10.1016/j.jadohealth.2005.10.008 16781977

[24] Hove MJ Risen JL. It's all in the timing: interpersonal synchrony increases affiliation. Soc Cogn. (2009) 27:949–60. doi: 10.1521/soco.2009.27.6.949

[25] Ryan RM Deci EL. Self-determination theory and the facilitation of intrinsic motivation, social development, and well-being. Am Psychol. (2000) 55:68–78. doi: 10.1037//0003-066X.55.1.68 11392867

[26] FredricksonBL. The role of positive emotions in positive psychology: the broaden-and-build theory of positive emotions. Am Psychol. (2001) 56:218–26. doi: 10.1037//0003-066X.56.3.21811315248 PMC3122271

[27] Bandura A. Self-efficacy: toward a unifying theory of behavioral change. Psychol Rev. (1977) 84:191–215. doi: 10.1037/0033-295X.84.2.191 847061

[28] Csikszentmihalyi M. Flow: The Psychology of Optimal Experience. New York, NY: Harper & Row (1990).

[29] Siegel DJ. The Developing Mind: How Relationships and the Brain Interact to Shape Who We Are. 2nd ed. New York: Guilford Press (2012).

[30] Bronfenbrenner U. Ecological models of human development. In:Husen T Postlethwaite TN, editors. International Encyclopedia of Education. Oxford: Elsevier (1994). p. 1643–7.

